# RACE AND ETHNIC DIFFERENCES IN PHYSICAL ACTIVITY, OSTEOPENIA, AND OSTEOPOROSIS: RESULTS FROM NHANES

**DOI:** 10.1093/geroni/igac059.358

**Published:** 2022-12-20

**Authors:** Elizabeth Vasquez, Md Towfiqul Alam, Rosenda Murillo

**Affiliations:** Albany University (SUNY), Rensselaer, New York, United States; University of North Carolina at Greensboro, Greensboro, North Carolina, United States; University of Houston, Houston, Texas, United States

## Abstract

**Introduction:**

Osteopenia and osteoporosis are common age-related disorders with enormous health and economic consequences to older adults and society. Physical activity (PA) is an important modifiable risk factor for bone mineral density (BMD). This study aims to determine whether current physical activity is related to osteopenia and osteoporosis (based on BMD) in a racial/ethnically diverse sample of older adults.

**Methods:**

Femoral bone BMD data from the National Health and Nutrition Examination Survey (NHANES 2009-2010, 2013-2014, 2017-2018) was obtained for 3,331 adults 60-80 years old. Self-reported PA was categorized into high, moderate, and low. Linear regression models that accounted for the complex survey design of NHANES examined the association between PA and BMD for each race/ethnic group.

**Results:**

Non-Latino blacks (blacks) and Latinos reported low levels of PA when compared to Non-Latino whites (whites) (40.7%, 38.2% and 32.4% respectively; p< 0.0001). Further, blacks and Latinos had a lower prevalence of osteoporosis (5.6%, 6.4% and 9.0% respectively; p< 0.0001), but have similar prevalence of normal BMD and osteopenia categories when compared to whites. There was a 0.03 g/cm2 difference in BMD between those in the high PA versus the low PA (p< 0.0001).

**Conclusion:**

Our findings indicate that despite lower levels of activity, black and Latino older adults were less likely to have osteoporosis. High levels of activity were significantly associated with higher BMD after controlling for confounders. Considering the prevalence and burden of osteopenia and osteoporosis and projected increases of the older population we need more research evidence supporting the role of PA.

